# Medication adherence, treatment satisfaction, and patient–doctor relationship in patients with mood disorders at a Saudi tertiary care hospital: a cross-sectional study

**DOI:** 10.3389/fpsyt.2025.1695769

**Published:** 2026-01-16

**Authors:** Ahmad H. Almadani, Ayedh H. Alghamdi, Mohammed A. Alkathiri, Fay H. AlBuqami, Saleh A. Alrahmah, Khalaf A. Aljumah, Ziyad B. Alenazi, Elaaf A. Albadi, Yara A. Alfarraj, Noorah E. Alkhilaiwi, Abdullah K. Muhnna, Mohammed A. Aljaffer

**Affiliations:** 1 Department of Psychiatry, College of Medicine, King Saud University, Riyadh, Saudi Arabia; 2 Department of Psychiatry, King Saud University Medical City, King Saud University, Riyadh, Saudi Arabia; 3 SABIC Psychological Health Research and Applications Chair (SPHRAC), Department of Psychiatry, College of Medicine, King Saud University, Riyadh, Saudi Arabia; 4 Department of Family and Community Medicine, King Saud University Medical City, King Saud University, Riyadh, Saudi Arabia; 5 College of Medicine, King Saud University, Riyadh, Saudi Arabia; 6 ERADAH Complex for Mental Health, Riyadh, Saudi Arabia

**Keywords:** medication adherence, mood disorders, patient–doctor relationship, Saudi Arabia, treatment satisfaction

## Abstract

**Introduction:**

Mood disorders are highly prevalent and associated with substantial impairment. This study examines medication adherence (MA), treatment satisfaction (TS), and patient–doctor relationships (PDRs) among adult patients with mood disorders at a Saudi tertiary hospital.

**Methods:**

This cross-sectional study’s tool employed a questionnaire developed by the researchers, along with the Arabic versions of the Medication Adherence Rating Scale, the Treatment Satisfaction Questionnaire for Medication (TSQM), and the Patient–Doctor Relationship Questionnaire (PDRQ).

**Results:**

Among the 260 participants, 79.2% demonstrated good MA, 34.23% exhibited low TS, and 62.31% reported good PDRs. Good MA was significantly associated with higher global TSQM scores (adjusted odds ratio [AOR] = 1.04; 95% confidence interval [CI]: 1.02–1.06; *p* < 0.001) and better PDR (*p* = 0.034). Illness duration was a significant factor associated with MA (AOR = 0.14; 95% CI: 0.02–0.86; *p* = 0.034 for 1–5 years compared with < 1 year), TS (AOR = 6.07; 95% CI, 1.80–20.48; *p* = 0.004 for > 10 years compared with < 1 year), and PDR (*p* = 0.028). Being supported by family and friends “to some extent” was associated with lower TS (AOR = 0.52; 95% CI: 0.30–0.92; *p* = 0.024) and poorer PDR (AOR = 0.37; 95% CI: 0.19–0.70; *p* = 0.002) compared with those receiving more support. Education level was significantly associated with TS, specifically for participants with a bachelor’s degree compared with uneducated participants (AOR = 5.04; 95% CI: 1.73–14.66; *p* = 0.003). MA was significantly related to age (AOR = 29.83; 95% CI: 1.75–508.3; *p* = 0.019 for > 65 years old compared with 18–25 years old) and number of regular medications (AOR = 0.66; 95% CI: 0.44–0.99; *p* = 0.044). A significant relationship was also observed between TS and the perception of having a mental illness and needing treatment (*p* = 0.001). PDR was significantly better among participants did not have to buy their own psychiatric medication compared with those who always purchased them (AOR = 5.04; 95% CI: 1.81–14.06; *p* = 0.002) and among those with a history of using alternative methods compared with those without (AOR = 2.23; 95% CI: 1.21–4.11; *p* = 0.01).

**Conclusion:**

Our findings highlight the interconnectedness of MA, TS, and PDR, as well as the influence of several factors. Patient-centered specific strategies are needed to address these factors.

## 1 Introduction

Mood disorders, also known as affective disorders, are a significant and prevalent category of mental illnesses (1). The Diagnostic and Statistical Manual of Mental Disorders (Fifth Edition) classifies these disorders into depressive, bipolar, and related disorders (1). They are associated with impairments in interpersonal, social, and occupational functioning (2). According to the 2019 Global Burden of Disease report, depressive disorders ranked as the second leading cause of years lived with disability and the 13th leading cause of disability-adjusted life-years (3). A systematic review and meta-analysis reported a pooled lifetime prevalence of approximately 9.6% (4). In Saudi Arabia, the Saudi National Mental Health Survey estimated a lifetime prevalence of approximately 9.3% for mood disorders (5). Despite the availability of effective treatments, it is estimated that around half of patients with depression receive no treatment, and most fail to adhere to their prescribed medication regimens (6–8). Moreover, nearly half of patients discontinue antidepressants within 6 months (9). Another systematic review and meta-analysis reported medication nonadherence rates of 44% for bipolar disorder and 50% for major depression (10).

The World Health Organization (WHO) defines adherence as “the extent to which a person’s behavior, taking medication, following a diet, and/or executing lifestyle changes, corresponds with agreed recommendations from a healthcare provider” (11). Research has indicated that medication nonadherence is a critical factor in translating treatment efficacy from research settings into clinical practice (12). In bipolar disorder, treatment nonadherence undermines medication efficacy and increases the risk of adverse outcomes (12). In depression, nonadherence can exacerbate symptom severity, diminish treatment response and remission rates, and increase the risk of relapse, recurrence, emergency department visits, and hospitalizations (13). Furthermore, a significant proportion of medication nonadherence remains undetected in clinical practice, with clinicians often underestimating its occurrence (14–16). Nonadherence presents a persistent clinical challenge that has been studied extensively to help clinicians prevent it (16). Factors affecting adherence can be grouped into demographics, symptom-related, medication-related, patient–doctor relationship (PDR), and comorbidity-related aspects (16).

Treatment satisfaction (TS), a key patient-reported outcome, has been consistently correlated with medication adherence (MA) in mood disorders (17–19) and significantly influences patients’ health-related decisions (20). However, nearly half of patients with chronic conditions make medication changes without consulting their physicians, often contributing to nonadherence (20). Perceived treatment effectiveness and convenience are major determinants of satisfaction (21), and medications that effectively alleviate mood symptoms are more likely to promote adherence (22–26). The PDR is also closely linked to MA, as shown in a study by García et al. (27). Adherence is a dynamic process that evolves through collaboration, strengthens PDR, and positively influences TS (28). These components are conceptually interconnected within behavioral frameworks, such as the health belief model, which proposes that health-related actions are shaped by perceived illness severity and susceptibility, as well as treatment-related benefits, barriers, and cues to action (29). Within this framework, TS reflects patients’ evaluations of treatment benefits and barriers, whereas PDR contributes to cues to action and the trust necessary for sustained adherence.

Regional evidence, particularly from Saudi Arabia, remains limited regarding MA, TS, and PDR collectively in individuals with mood disorders. Studies in the region have generally examined these variables in isolation, highlighting a gap in understanding how they interact within local cultural and clinical contexts (30, 31). Addressing this gap is essential for informing practice and service delivery. Accordingly, the present study investigated MA, TS, and PDR among adult patients with mood disorders at a Saudi tertiary hospital.

## 2 Materials and methods

### 2.1 Study design, setting, and participants

We conducted a quantitative cross-sectional study among patients aged 18 and above with mood disorders attending psychiatric clinics at King Khalid University Hospital (KKUH) in Riyadh, Saudi Arabia. We excluded those younger than 18 years, those with communication barriers, and those with secondary mood disorders (for example, those secondary to medications, substances, or medical conditions). The size of the target population, based on information obtained from KKUH’s Information Technology (IT) Department, was 1,097 after applying the inclusion and exclusion criteria. The IT Department used several keywords to identify the target population, including “depressive”, “bipolar”, “mood (affective) disorder”, “depression”, “dysthymia”, “cyclothymia”, “mania with psychotic symptoms”, “mania without psychotic symptoms”, and “hypomania”. Using the Raosoft sample size calculator (http://www.raosoft.com/samplesize.html) with a 5% margin of error and a 95% confidence level, the required sample size was estimated to be 285 based on the target population. After obtaining the list of eligible participants from the IT Department in a Microsoft Excel spreadsheet, a random number was assigned to each participant using the Excel function =RAND(), and the list was sorted accordingly. Recruitment began from the top of the randomized list, inviting participants sequentially. If a participant could not be reached or declined participation, the next participant on the list was contacted. This procedure ensured simple random sampling from the target population.

### 2.2 Study instrument

The study tool (an online electronic survey) was sent to the participants via WhatsApp/SMS after we contacted them by telephone to explain the research and invite their participation. Data were collected between the end of December 2024 and early April 2025.

The study instrument, delivered in Arabic, consisted of four sections: a self-developed questionnaire and the Arabic versions of the Medication Adherence Rating Scale (MARS-10), the Treatment Satisfaction Questionnaire for Medication (TSQM-1.4), and the Patient–Doctor Relationship Questionnaire (PDRQ-9).

The developed questionnaire assesses sociodemographic, psychiatric, and medical-related information. It includes questions about age, gender, marital status, living arrangement (i.e., alone or with others), level of education, and employment status; psychiatric diagnosis(es), duration of the mood disorder, number of psychiatric hospitalizations, and degree of insight into the mental illness and need for treatment; use of illegal substances; number of regular medications for the psychiatric illness and ease of obtaining them; payment for psychotropic medication; feelings of shame about taking psychotropic medication and whether this stigma affects adherence; perceived support from others regarding the mental condition; chronic medical illness(es); and alternative (i.e., nonmedication) treatment modalities tried for the psychiatric illness (including what they are, whether used alone or with medication, and the belief about their effectiveness compared with the prescribed psychotropic medication).

MARS-10 is a 10-item dichotomous (yes/no) self-report tool used to assess MA behavior (32). The total score ranges from 0 to 10 (32). The score is calculated by considering that a compliant patient would answer “No” to questions 1–6 and 9–10 and “Yes” to questions 7 and 8 (33). A cutoff score of six or higher was deemed indicative of MA (33–36). Regarding validity and reliability, Fialko et al. (2008) reported that MARS-10 demonstrated an internal consistency of 0.60 and a 12-month test–retest reliability of 0.52 (32). In the Arabic translation of MARS-10, Cronbach’s α coefficient was 0.89 in a study conducted in secondary and tertiary care clinics (37). Permission to use MARS-10 was obtained from the corresponding author of the Arabic translation.

TSQM-1.4 is a validated and reliable instrument for assessing satisfaction with medication in both clinical practice and research (38–40). The scale comprises four key domains of medication satisfaction: effectiveness (items 1–3), side effects (items 4–8), convenience (items 9–11), and global satisfaction (items 12–14). Domain scores were calculated according to the guidelines provided by the instrument’s authors (40). Each domain is scored on a scale from 0 to 100, with higher scores reflecting greater satisfaction. In our study, TS was assessed using the global satisfaction score, categorized as follows: scores above 90 indicate very high satisfaction, 80–89 high, 65–79 moderate, 40–64 low, and below 40 very low satisfaction (41). TSQM-1.4 demonstrated a Cronbach’s α coefficient above 0.86 in a study involving patients with cystic fibrosis (42) and 0.85 in a study of chronic diseases (39). Additionally, the Arabic translation of TSQM-1.4 showed a Cronbach’s α coefficient greater than 0.75 in a study on anticoagulant therapy (43). We obtained permission to use TSQM-1.4 from the corresponding author of the Arabic translation.

PDRQ-9 is used to assess the quality of the PDR from the patient’s perspective (44). It comprises nine items designed to measure satisfaction with the PDR. PDRQ-9 has demonstrated high reliability and validity in primary care (45). Item scores range from 1 (extremely dissatisfied) to 5 (extremely satisfied), yielding a total score between 9 and 45 (46). We used the following cutoff values based on previous studies: 17 or lower (poor PDR), 18–35 (moderate relationship), and 36 or higher (good relationship), based on previous studies (47, 48). PDRQ-9 demonstrated a Cronbach’s α coefficient of 0.96 in a primary-care study and 0.92 in a study of schizophrenia (45, 49). Additionally, the Arabic translation of PDRQ-9 had a Cronbach’s α coefficient of 0.979 in a representative cross-sectional Egyptian population survey (50). Permission to use PDRQ-9 was obtained from the corresponding author of the Arabic translation.

### 2.3 Data analysis

Data were analyzed using the statistical software SPSS version 24.0 (IBM Inc., Chicago, IL, USA). Descriptive statistics (frequencies, percentages, mean, and standard deviation) were used to describe the categorical and quantitative variables. Univariate analysis was conducted using Student’s *t*-test for independent samples and one-way analysis of variance, followed by *post-hoc* test for quantitative outcome variables to compare mean values across categorical variables with two or more groups. Normality of continuous variables was evaluated using the Shapiro–Wilk test. Pearson’s Chi-square test was used to assess the association between categorical and outcome variables. Odds ratios were calculated to measure the strength of associations. If the Chi-square test (for a 2 × 2 table) was not applicable, Fisher’s exact test was used. Multicollinearity was assessed using variance inflation factors (VIF), with values > 5 indicating potential concern. Logistic model adequacy was evaluated using Nagelkerke *R*², the Akaike Information Criterion (AIC), and the Hosmer–Lemeshow goodness-of-fit test. A *p*-value ≤ 0.05 and 95% confidence intervals were used to report statistical significance and precision of the results.

### 2.4 Ethical considerations

This study was approved by the Institutional Review Board of the College of Medicine at King Saud University (KSU), Riyadh, Saudi Arabia (Research Project No. E-24-8962). Participants read the informed-consent statement and then selected “Next” to access the study’s survey. They were provided with explanations regarding confidentiality, data anonymity, the study’s scope, and the principal investigator’s contact information. The right to participate was granted by clicking the link to provide informed consent.

## 3 Results

Of the 450 individuals contacted, 272 began the survey, regardless of whether they completed it, yielding a response rate of 60.4%. Of these, 260 provided complete data, resulting in a completion rate of 95.6%. Consequently, the study included 260 patients with mood disorders, predominantly women (68.85%) and diagnosed with depressive disorders (79.62%). Nearly half of the participants were married (46.54%) and living with others (96.15%). Most patients (72.69%) had never required psychiatric hospitalization, and approximately two-thirds acknowledged having a mental illness requiring treatment. Detailed sociodemographic, psychiatric, and medical characteristics are presented in **Table 1**.

**Table 1:** Sociodemographic, psychiatric, and medical-related data of the studied patients.

Item	Total patients (*n =* 260)
Age (years)	
18–25	47 (18.08%)
26–35	49 (18.85%)
36–45	40 (15.38%)
46–55	61 (23.46%)
56–65	41 (15.77%)
> 65	22 (8.46%)
Gender	
Men	81 (31.15%)
Women	179 (68.85%)
Marital status	
Single	88 (33.85%)
Married	121 (46.54%)
Divorced/Separated	32 (12.31%)
Widowed	19 (7.31%)
Living condition	
Alone	10 (3.85%)
Living with family, friends, a roommate, etc.	250 (96.15%)
Education level	
Uneducated	32 (12.31%)
High school or below	82 (31.54%)
Bachelor’s degree	126 (48.46%)
Master’s or PhD	20 (7.69%)
Employment status	
Unemployed	155 (59.62%)
Employed	72 (27.69%)
Retired	33 (12.69%)
Mood disorder	
Depressive	207 (79.62%)
Bipolar	53 (20.38%)
Other psychiatric disorders^a^	(*n* = 52)
Anxiety	41 (78.85%)
OCD	8 (15.38%)
BPD	3 (5.77%)
Psychosis	5 (9.61%)
Duration of mood disorder (years)	
< 1	16 (6.15%)
1–5	81 (31.15%)
6–10	44 (16.92%)
> 10	119 (45.77%)
Number of psychiatric ward admissions	
0	189 (72.69%)
1	34 (13.08%)
2–3	20 (7.69%)
> 3	17 (6.54%)
To what extent do you think you have a mental illness and need treatment?	
I’m certain.	169 (65%)
I’m somewhat certain.	52 (20%)
I don’t think I have a mental illness.	39 (15%)
Have you ever used any illegal substances/drugs (for example, alcohol and marijuana)?	
No	241 (92.69%)
Yes	19 (7.31%)
Number of regular medications (taken regularly, not as needed) for your mental health conditions	
0	23 (8.85%)
1–2	207 (79.62%)
3–4	26 (10%)
≥ 5	4 (1.54%)
How easy is it for you to get your regular medication for your mental health conditions?	(*n* = 237)
Always easy	107 (45.15%)
Easy most of the time	88 (37.13%)
Always difficult	22 (9.28%)
Difficult most of the time	20 (8.44%)
Do you have to buy your own psychiatric medication?	
Yes, always	31 (11.92%)
Yes, sometimes	151 (58.08%)
Never	78 (30%)
Do you feel shame about taking psychiatric medication?	
No	209 (80.38%)
Yes	51 (19.62%)
If yes, do you think that your feeling of stigma affects your adherence to taking your psychiatric medication regularly?	(*n* = 51)
No	35 (68.63%)
Yes	16 (31.37%)
To what extent do you feel supported by family and friends regarding your mental illness?	
I feel very supported.	125 (48.08%)
I feel supported to some extent.	98 (37.69%)
I don’t feel supported.	37 (14.23%)
Chronic diseases (such as hypertension and diabetes)	
No	132 (50.77%)
Yes	128 (49.23%)
Have you tried using alternative methods to treat your mental illness?	
No	115 (44.23%)
Yes	145 (55.77%)
Alternative methods used^a^	(*n* = 145)
Ruqiyah	140 (96.55%)
Cauterization	10 (6.9%)
Herbs	38 (26.21%)
Other (religious practices, vitamins, minerals, dietary habits, etc.)	10 (6.9%)
Have you tried alternative methods alone or with psychiatric medication?	(*n* = 145)
Alone	44 (30.34%)
With psychiatric medication	101 (69.66%)
Do you think alternative methods are more effective than psychiatric medication?	(*n* = 145)
No	109 (75.17%)
Yes	36 (24.83%)

Numerical data are presented as mean ± SD, categorical data as frequency (%).

*OCD*, obsessive–compulsive disorder; *BPD*, borderline personality disorder.

a More than one answer is allowed.

Overall, 79.2% of participants demonstrated good MA (MARS-10 score ≥ 6), with a mean total score of 6.96 ± 2.05. The MARS-10 subscale responses indicated that most patients recognized the benefits of their medication, with over 80% reporting clearer thoughts and believing that continued medication use helps prevent illness recurrence (**Supplementary Table S1**).

Bivariate analyses (**Table 2**) identified several factors significantly associated with adherence. Younger patients (18–25 years old) and single individuals were more prevalent in the poor-adherence group. Additionally, patients with poor adherence were more likely to use illegal substances (*p* = 0.003), experience medication-related shame (*p* < 0.001), and perceive alternative treatments as superior to psychiatric medication (*p* = 0.044).

**Table 2:** Relationship between patients’ sociodemographic, psychiatric, and medical-related data and medication adherence according to MARS-10 score.

Item	Adherence		*p*-value
	Poor (*n* = 54)	Good (*n* = 206)	
Age (years)			
18–25	18 (33.33%)	29 (14.08%)	0.028
26–35	9 (16.67%)	40 (19.42%)	
36–45	6 (11.11%)	34 (16.5%)	
46–55	13 (24.07%)	48 (23.3%)	
56–65	6 (11.11%)	35 (16.99%)	
> 65	2 (3.7%)	20 (9.71%)	
Gender			
Men	18 (33.33%)	63 (30.58%)	0.698
Women	36 (66.67%)	143 (69.42%)	
Marital status			
Single	27 (50%)	61 (29.61%)	0.037
Married	19 (35.19%)	102 (49.51%)	
Divorced/Separated	4 (7.41%)	28 (13.59%)	
Widow	4 (7.41%)	15 (7.28%)	
Living condition			
Alone	2 (3.7%)	8 (3.88%)	0.951
Living with family, friends, a roommate, etc.	52 (96.3%)	198 (96.12%)	
Education level			
Uneducated	4 (7.41%)	28 (13.59%)	0.304
High school or below	22 (40.74%)	60 (29.13%)	
Bachelor’s degree	25 (46.3%)	101 (49.03%)	
Master’s or PhD	3 (5.56%)	17 (8.25%)	
Employment status			
Unemployed	36 (66.67%)	119 (57.77%)	0.487
Employed	12 (22.22%)	60 (29.13%)	
Retired	6 (11.11%)	27 (13.11%)	
Mood disorder			
Depressive	42 (77.78%)	165 (80.1%)	0.706
Bipolar	12 (22.22%)	41 (19.9%)	
Duration of illness (years)			
< 1	3 (5.56%)	13 (6.31%)	0.185
1–5	22 (40.74%)	59 (28.64%)	
6–10	11 (20.37%)	33 (16.02%)	
> 10	18 (33.33%)	101 (49.03%)	
Number of psychiatric ward admissions			
0	36 (66.67%)	153 (74.27%)	0.120
1	8 (14.81%)	26 (12.62%)	
2–3	8 (14.81%)	12 (5.83%)	
> 3	2 (3.7%)	15 (7.28%)	
To what extent do you think you have a mental illness and need treatment?			
I’m certain.	31 (57.41%)	138 (66.99%)	0.353
I’m somewhat certain.	12 (22.22%)	40 (19.42%)	
I don’t think I have a mental illness.	11 (20.37%)	28 (13.59%)	
Have you ever used any illegal substances/drugs (for example, alcohol and marijuana)?			
No	45 (83.33%)	196 (95.15%)	0.003
Yes	9 (16.67%)	10 (4.85%)	
Number of regular medications (taken constantly, not as needed) for your mental health conditions			
1–2	40 (74.07%)	167 (81.07%)	0.134
3–4	4 (7.41%)	22 (10.68%)	
≥ 5	1 (1.85%)	3 (1.46%)	
0	9 (16.67%)	14 (6.8%)	
How easy is it for you to get your regular medication for your mental health conditions?	(*n* = 45)	(*n* = 192)	
Always easy	13 (28.89%)	94 (48.96%)	0.112
Easy most of the time	22 (48.89%)	66 (34.38%)	
Always difficult	5 (11.11%)	17 (8.85%)	
Difficult most of the time	5 (11.11%)	15 (7.81%)	
Do you have to buy your own psychiatric medication?			
Yes, always	4 (7.41%)	27 (13.11%)	0.404
Yes, sometimes	35 (64.81%)	116 (56.31%)	
Never	15 (27.78%)	63 (30.58%)	
Do you feel shame about taking psychiatric medication?			
No	33 (61.11%)	176 (85.44%)	< 0.001
Yes	21 (38.89%)	30 (14.56%)	
If yes, do you think that your feeling of stigma affects your adherence to taking your psychiatric medication regularly?	(*n* = 21)	(*n* = 30)	
No	8 (38.1%)	27 (90%)	< 0.001
Yes	13 (61.9%)	3 (10%)	
To what extent do you feel supported by family and friends regarding your mental illness?			
I feel very supported.	18 (33.3%)	107 (51.9%)	0.051
I feel supported to some extent.	26 (48.1%)	72 (35%)	
I don’t feel supported.	10 (18.5%)	27 (13.1%)	
Chronic diseases (such as hypertension and diabetes)			
No	29 (53.7%)	103 (50%)	0.628
Yes	25 (46.3%)	103 (50%)	
Have you tried using alternative methods to treat your mental illness?			
No	23 (42.59%)	92 (44.66%)	0.785
Yes	31 (57.41%)	114 (55.34%)	
Have you tried alternative methods alone or with psychiatric medication?	(*n* = 31)	(*n* = 114)	
Alone	12 (38.7%)	32 (28.1%)	0.253
With psychiatric medication	19 (61.3%)	82 (71.9%)	
Do you think alternative methods are more effective than psychiatric medication?	(*n* = 31)	(*n* = 114)	
No	19 (61.29%)	90 (78.95%)	0.044
Yes	12 (38.71%)	24 (21.05%)	

Numerical data are presented as mean ± SD, categorical data as frequency (%). Statistical significance was set at *p*-value < 0.05.

The TS levels varied considerably across the sample, with approximately one-third (34.23%) reporting low satisfaction and just over one-quarter (26.92%) expressing very high satisfaction. The mean global satisfaction score was 69.69 ± 24.52. Domain-specific analyses indicated relatively higher satisfaction with medication convenience (mean score = 76.18 ± 19.44) compared with side effects (mean score = 41.71 ± 21.71), with half of the participants experiencing some adverse effects. Complete TSQM-1.4 domain results are provided in **Supplementary Table S2**.

Several factors demonstrated significant relationships with satisfaction levels (**Table 3**). A clear dose–response pattern emerged between illness insight and satisfaction, with the proportion of patients certain about their mental illness increasing progressively from the very low (45.83%) to the very high (80%) satisfaction groups (*p* = 0.001). Similarly, strong family and social support was associated with higher satisfaction (*p* = 0.003), whereas perceiving alternative methods as more effective than psychiatric medication was strongly associated with lower satisfaction (*p* < 0.001).

**Table 3:** Relationship between patients’ sociodemographic, psychiatric, and medical-related data and global satisfaction with treatment according to the TSQM-1.4 global score.

Item	Satisfaction					*p*-value
	Very low (*n =* 24)	Low (*n* = 89)	Moderate (*n* = 56)	High (*n* = 21)	Very high (*n* = 70)	
Age (years)						
18–25	6 (25%)	17 (19.1%)	10 (17.86%)	4 (19.05%)	10 (14.29%)	0.727
26–35	4 (16.67%)	22 (24.72%)	6 (10.71%)	3 (14.29%)	14 (20%)	
36–45	5 (20.83%)	13 (14.61%)	9 (16.07%)	4 (19.05%)	9 (12.86%)	
46–55	3 (12.5%)	23 (25.84%)	13 (23.21%)	6 (28.57%)	16 (22.86%)	
56–65	4 (16.67%)	7 (7.87%)	12 (21.43%)	4 (19.05%)	14 (20%)	
> 65	2 (8.33%)	7 (7.87%)	6 (10.71%)	0 (0%)	7 (10%)	
Gender						
Men	7 (29.17%)	30 (33.71%)	23 (41.07%)	5 (23.81%)	16 (22.86%)	0.226
Women	17 (70.83%)	59 (66.29%)	33 (58.93%)	16 (76.19%)	54 (77.14%)	
Marital status						
Single	11 (45.83%)	37 (41.57%)	14 (25%)	7 (33.33%)	19 (27.14%)	0.229
Married	7 (29.17%)	40 (44.94%)	31 (55.36%)	10 (47.62%)	33 (47.14%)	
Divorced/Separated	4 (16.67%)	5 (5.62%)	6 (10.71%)	3 (14.29%)	14 (20%)	
Widow	2 (8.33%)	7 (7.87%)	5 (8.93%)	1 (4.76%)	4 (5.71%)	
Living condition						
Alone	1 (4.17%)	5 (5.62%)	4 (7.14%)	0 (0%)	0 (0%)	0.196
Living with family, friends, a roommate, etc.	23 (95.83%)	84 (94.38%)	52 (92.86%)	21 (100%)	70 (100%)	
Education level						
Uneducated	4 (16.67%)	9 (10.11%)	5 (8.93%)	3 (14.29%)	11 (15.71%)	0.664
High school or below	8 (33.33%)	29 (32.58%)	15 (26.79%)	8 (38.1%)	22 (31.43%)	
Bachelor’s degree	9 (37.5%)	43 (48.31%)	32 (57.14%)	7 (33.33%)	35 (50%)	
Master’s or PhD	3 (12.5%)	8 (8.99%)	4 (7.14%)	3 (14.29%)	2 (2.86%)	
Employment status						
Unemployed	16 (66.67%)	52 (58.43%)	27 (48.21%)	11 (52.38%)	49 (70%)	0.421
Employed	6 (25%)	24 (26.97%)	19 (33.93%)	7 (33.33%)	16 (22.86%)	
Retired	2 (8.33%)	13 (14.61%)	10 (17.86%)	3 (14.29%)	5 (7.14%)	
Mood disorder						
Depressive	17 (70.83%)	71 (79.78%)	44 (78.57%)	19 (90.48%)	56 (80%)	0.607
Bipolar	7 (29.17%)	18 (20.22%)	12 (21.43%)	2 (9.52%)	14 (20%)	
Duration of illness (years)						
< 1	2 (8.33%)	10 (11.24%)	4 (7.14%)	0 (0%)	0 (0%)	0.168
1–5	10 (41.67%)	28 (31.46%)	17 (30.36%)	7 (33.33%)	19 (27.14%)	
6–10	5 (20.83%)	16 (17.98%)	10 (17.86%)	3 (14.29%)	10 (14.29%)	
> 10	7 (29.17%)	35 (39.33%)	25 (44.64%)	11 (52.38%)	41 (58.57%)	
Number of psychiatric ward admissions						
0	14 (58.33%)	67 (75.28%)	45 (80.36%)	17 (80.95%)	46 (65.71%)	0.219
1	3 (12.5%)	13 (14.61%)	3 (5.36%)	2 (9.52%)	13 (18.57%)	
2–3	5 (20.83%)	6 (6.74%)	4 (7.14%)	1 (4.76%)	4 (5.71%)	
> 3	2 (8.33%)	3 (3.37%)	4 (7.14%)	1 (4.76%)	7 (10%)	
To what extent do you think you have a mental illness and need treatment?						
I’m certain.	11 (45.83%)	49 (55.06%)	38 (67.86%)	15 (71.43%)	56 (80%)	0.001
I’m somewhat certain.	3 (12.5%)	27 (30.34%)	11 (19.64%)	3 (14.29%)	8 (11.43%)	
I don’t think I have a mental illness.	10 (41.67%)	13 (14.61%)	7 (12.5%)	3 (14.29%)	6 (8.57%)	
Have you ever used any illegal substances/drugs (for example, alcohol and marijuana)?						
No	22 (91.67%)	80 (89.89%)	52 (92.86%)	19 (90.48%)	68 (97.14%)	0.513
Yes	2 (8.33%)	9 (10.11%)	4 (7.14%)	2 (9.52%)	2 (2.86%)	
Number of regular medications (taken constantly, not as needed) for your mental health conditions						
1–2	16 (66.67%)	72 (80.9%)	41 (73.21%)	18 (85.71%)	60 (85.71%)	0.125
3–4	3 (12.5%)	6 (6.74%)	7 (12.5%)	2 (9.52%)	8 (11.43%)	
≥ 5	0 (0%)	1 (1.12%)	3 (5.36%)	0 (0%)	0 (0%)	
0	5 (20.83%)	10 (11.24%)	5 (8.93%)	1 (4.76%)	2 (2.86%)	
How easy is it for you to get your regular medication for your mental health conditions?	(*n* = 19)	(*n* = 79)	(*n* = 51)	(*n* = 20)	(*n* = 68)	
Always easy	7 (36.84%)	27 (34.18%)	23 (45.1%)	7 (35%)	43 (63.24%)	0.066
Easy most of the time	8 (42.11%)	33 (41.77%)	20 (39.22%)	10 (50%)	17 (25%)	
Always difficult	3 (15.79%)	10 (12.66%)	2 (3.92%)	1 (5%)	6 (8.82%)	
Difficult most of the time	1 (5.26%)	9 (11.39%)	6 (11.76%)	2 (10%)	2 (2.94%)	
Do you have to buy your own psychiatric medication?						
Yes, always	2 (8.33%)	12 (13.48%)	4 (7.14%)	3 (14.29%)	10 (14.29%)	0.169
Yes, sometimes	13 (54.17%)	58 (65.17%)	37 (66.07%)	12 (57.14%)	31 (44.29%)	
Never	9 (37.5%)	19 (21.35%)	15 (26.79%)	6 (28.57%)	29 (41.43%)	
Do you feel shame about taking psychiatric medication?						
No	18 (75%)	64 (71.91%)	45 (80.36%)	18 (85.71%)	64 (91.43%)	0.036
Yes	6 (25%)	25 (28.09%)	11 (19.64%)	3 (14.29%)	6 (8.57%)	
If yes, do you think that your feeling of stigma affects your adherence to taking your psychiatric medication regularly?	(*n* = 6)	(*n* = 25)	(*n* = 11)	(*n* = 3)	(*n* = 6)	
No	2 (33.33%)	17 (68%)	9 (81.82%)	3 (100%)	4 (66.67%)	0.219
Yes	4 (66.67%)	8 (32%)	2 (18.18%)	0 (0%)	2 (33.33%)	
To what extent do you feel supported by family and friends regarding your mental illness?						
I feel very supported.	6 (25%)	37 (41.57%)	26 (46.43%)	7 (33.33%)	49 (70%)	0.003
I feel supported to some extent.	12 (50%)	40 (44.94%)	22 (39.29%)	9 (42.86%)	15 (21.43%)	
I don’t feel supported.	6 (25%)	12 (13.48%)	8 (14.29%)	5 (23.81%)	6 (8.57%)	
Chronic diseases (such as hypertension and diabetes)						
No	14 (58.33%)	49 (55.06%)	25 (44.64%)	12 (57.14%)	32 (45.71%)	0.541
Yes	10 (41.67%)	40 (44.94%)	31 (55.36%)	9 (42.86%)	38 (54.29%)	
Have you tried using alternative methods to treat your mental illness?						
No	13 (54.17%)	36 (40.45%)	30 (53.57%)	8 (38.1%)	28 (40%)	0.369
Yes	11 (45.83%)	53 (59.55%)	26 (46.43%)	13 (61.9%)	42 (60%)	
Have you tried alternative methods alone or with psychiatric medication?	(*n* = 11)	(*n* = 53)	(*n* = 26)	(*n* = 13)	(*n* = 42)	
Alone	5 (45.45%)	9 (16.98%)	8 (30.77%)	7 (53.85%)	15 (35.71%)	0.047
With psychiatric medication	6 (54.55%)	44 (83.02%)	18 (69.23%)	6 (46.15%)	27 (64.29%)	
Do you think alternative methods are more effective than psychiatric medication?	(*n* = 11)	(*n* = 53)	(*n* = 26)	(*n* = 13)	(*n* = 42)	
No	3 (27.27%)	36 (67.92%)	23 (88.46%)	12 (92.31%)	35 (83.33%)	< 0.001
Yes	8 (72.73%)	17 (32.08%)	3 (11.54%)	1 (7.69%)	7 (16.67%)	

Numerical data are presented as mean ± SD, categorical data as frequency (%). Statistical significance was set at *p*-value < 0.05.

The majority of participants (62.31%) reported good PDR (PDRQ-9 score ≥ 36), with a mean score of 36.5 ± 8.08. Most patients strongly endorsed positive aspects of their therapeutic relationships, including trust in their physician (49.23%). Full PDRQ-9 item responses are provided in **Supplementary Table S3**.

Education level, illness duration, medication-related shame, and social support showed significant associations with PDR quality (**Table 4**). Notably, longer illness duration was associated with better relationships (*p* = 0.028), and patients reporting strong family support were more likely to have good physician relationships (*p* = 0.002).

**Table 4:** Relation between patients’ sociodemographic, psychiatric, and medical-related data and patient–doctor relationship according to PDRQ-9 score.

Item	Patient–doctor relationship			*p*-value
	Poor (*n* = 7)	Moderate (*n* = 91)	Good (*n* = 162)	
Age (years)				
18–25	2 (28.57%)	18 (19.78%)	27 (16.67%)	0.377
26–35	4 (57.14%)	17 (18.68%)	28 (17.28%)	
36–45	1 (14.29%)	15 (16.48%)	24 (14.81%)	
46–55	0 (0%)	19 (20.88%)	42 (25.93%)	
56–65	0 (0%)	15 (16.48%)	26 (16.05%)	
> 65	0 (0%)	7 (7.69%)	15 (9.26%)	
Gender				
Men	1 (14.29%)	34 (37.36%)	46 (28.4%)	0.208
Women	6 (85.71%)	57 (62.64%)	116 (71.6%)	
Marital status				
Single	5 (71.43%)	33 (36.26%)	50 (30.86%)	0.231
Married	2 (28.57%)	45 (49.45%)	74 (45.68%)	
Divorced/Separated	0 (0%)	8 (8.79%)	24 (14.81%)	
Widow	0 (0%)	5 (5.49%)	14 (8.64%)	
Living condition				
Alone	0 (0%)	4 (4.4%)	6 (3.7%)	0.834
Living with family, friends, a roommate, etc.	7 (100%)	87 (95.6%)	156 (96.3%)	
Education level				
Uneducated	0 (0%)	6 (6.59%)	26 (16.05%)	0.038
High school or below	2 (28.57%)	22 (24.18%)	58 (35.8%)	
Bachelor’s degree	4 (57.14%)	53 (58.24%)	69 (42.59%)	
Master’s or PhD	1 (14.29%)	10 (10.99%)	9 (5.56%)	
Employment status				
Unemployed	5 (71.43%)	48 (52.75%)	102 (62.96%)	0.446
Employed	2 (28.57%)	30 (32.97%)	40 (24.69%)	
Retired	0 (0%)	13 (14.29%)	20 (12.35%)	
Mood disorder				
Depression	4 (57.14%)	73 (80.22%)	130 (80.25%)	0.327
Bipolar	3 (42.86%)	18 (19.78%)	32 (19.75%)	
Duration of illness (years)				
< 1	0 (0%)	4 (4.4%)	12 (7.41%)	0.028
1–5	2 (28.57%)	32 (35.16%)	47 (29.01%)	
6–10	4 (57.14%)	19 (20.88%)	21 (12.96%)	
> 10	1 (14.29%)	36 (39.56%)	82 (50.62%)	
Number of psychiatric ward admissions				
0	3 (42.86%)	69 (75.82%)	117 (72.22%)	0.183
1	2 (28.57%)	13 (14.29%)	19 (11.73%)	
2–3	2 (28.57%)	4 (4.4%)	14 (8.64%)	
> 3	0 (0%)	5 (5.49%)	12 (7.41%)	
To what extent do you think you have a mental illness and need treatment?				
I’m certain.	2 (28.57%)	54 (59.34%)	113 (69.75%)	0.082
I’m somewhat certain.	2 (28.57%)	21 (23.08%)	29 (17.9%)	
I don’t think I have a mental illness.	3 (42.86%)	16 (17.58%)	20 (12.35%)	
Have you ever used any illegal substances/drugs?				
No	6 (85.71%)	81 (89.01%)	154 (95.06%)	0.160
Yes	1 (14.29%)	10 (10.99%)	8 (4.94%)	
Number of regular medications (taken constantly, not as needed) for your mental health conditions				
1–2	4 (57.14%)	78 (85.71%)	125 (77.16%)	0.178
3–4	1 (14.29%)	4 (4.4%)	21 (12.96%)	
≥ 5	0 (0%)	1 (1.1%)	3 (1.85%)	
0	2 (28.57%)	8 (8.79%)	13 (8.02%)	
How easy is it for you to get your regular medication for your mental health conditions?	(*n* = 5)	(*n* = 83)	(*n* = 149)	
Always easy	3 (60%)	26 (31.33%)	78 (52.35%)	0.063
Easy most of the time	2 (40%)	36 (43.37%)	50 (33.56%)	
Always difficult	0 (0%)	12 (14.46%)	10 (6.71%)	
Difficult most of the time	0 (0%)	9 (10.84%)	11 (7.38%)	
Do you have to buy your own psychiatric medication?				
Yes, always	2 (28.57%)	14 (15.38%)	15 (9.26%)	0.092
Yes, sometimes	4 (57.14%)	57 (62.64%)	90 (55.56%)	
Never	1 (14.29%)	20 (21.98%)	57 (35.19%)	
Do you feel shame about taking psychiatric medication?				
No	6 (85.71%)	65 (71.43%)	138 (85.19%)	0.028
Yes	1 (14.29%)	26 (28.57%)	24 (14.81%)	
If yes, do you think that your feeling of stigma affects your adherence to taking your psychiatric medication regularly?	(*n* = 1)	(*n* = 26)	(*n* = 24)	
No	0 (0%)	17 (65.38%)	18 (75%)	0.251
Yes	1 (100%)	9 (34.62%)	6 (25%)	
To what extent do you feel supported by family and friends regarding your mental illness?				
I feel very supported.	2 (28.57%)	29 (31.87%)	94 (58.02%)	0.002
I feel supported to some extent.	4 (57.14%)	46 (50.55%)	48 (29.63%)	
I don’t feel supported.	1 (14.29%)	16 (17.58%)	20 (12.35%)	
Chronic diseases (such as hypertension and diabetes)				
No	5 (71.43%)	47 (51.65%)	80 (49.38%)	0.510
Yes	2 (28.57%)	44 (48.35%)	82 (50.62%)	
Have you tried using alternative methods to treat your mental illness?				
No	4 (57.14%)	48 (52.75%)	63 (38.89%)	0.081
Yes	3 (42.86%)	43 (47.25%)	99 (61.11%)	
Have you tried alternative methods alone or with psychiatric medication?	(*n* = 3)	(*n* = 43)	(*n* = 99)	
Alone	2 (66.67%)	11 (25.58%)	31 (31.31%)	0.305
With psychiatric medication	1 (33.33%)	32 (74.42%)	68 (68.69%)	
Do you think alternative methods are more effective than psychiatric medication?	(*n* = 3)	(*n* = 43)	(*n* = 99)	
No	1 (33.33%)	29 (67.44%)	79 (79.8%)	0.070
Yes	2 (66.67%)	14 (32.56%)	20 (20.2%)	

Numerical data are presented as mean ± SD, categorical data as frequency (%). Statistical significance was set at *p*-value < 0.05.

Good MA was significantly associated with higher TS (*p* < 0.001) and better PDR (*p* = 0.034). Furthermore, TS and PDR quality were strongly interrelated (*p* < 0.001), as shown in **Table 5**.

**Table 5:** Relationship between medication adherence, treatment satisfaction, and the patient–doctor relationship.

Item	Adherence		*p*-value	Satisfaction					*p*-value
	Poor	Good		Very low	Low	Moderate	High	Very high	
Satisfaction									
Very low	12 (22.22%)	12 (5.83%)	< 0.001						
Low	27 (50%)	62 (30.1%)							
Moderate	7 (12.96%)	49 (23.79%)							
High	1 (1.85%)	20 (9.71%)							
Very high	7 (12.96%)	63 (30.58%)							
Patient–doctor relationship									
Poor	3 (5.56%)	4 (1.94%)	0.034	4 (16.67%)	2 (2.25%)	0 (0%)	0 (0%)	1 (1.43%)	< 0.001
Moderate	25 (46.3%)	66 (32.04%)		12 (50%)	45 (50.56%)	21 (37.5%)	3 (14.29%)	10 (14.29%)	
Good	26 (48.15%)	136 (66.02%)		8 (33.33%)	42 (47.19%)	35 (62.5%)	18 (85.71%)	59 (84.29%)	

Numerical data are presented as mean ± SD, categorical data as frequency (%). Statistical significance was set at *p*-value < 0.05.

Multivariable logistic regression analysis (**Table 6**) identified several independent correlates of MA. Older age groups exhibited substantially higher odds of adherence compared with the youngest group (18–25 years old), with adjusted odds ratios (ORs) of 7.65 (95% confidence interval [CI]: 1.19–49.19; *p* = 0.032) for ages 36–45, 11.01 (95% CI: 1.02–119.46; *p* = 0.049) for ages 56–65, and 29.83 (95% CI: 1.75–508.3; *p* = 0.019) for those over 65 years. Conversely, intermediate illness durations (1–5 and 6–10 years) were associated with significantly lower odds of adherence (OR = 0.14, 95% CI: 0.02–0.86; *p* = 0.034 for both groups) compared with an illness duration of less than 1 year.

**Table 6:** Multiple logistic regression analysis for factors associated with medication adherence.

Item	Multivariable analysis		
	Adjusted OR	95% CI	*p*-value
Age (years)			
18–25	Ref		
26–35	3.69	0.91–14.9	0.067
36–45	7.65	1.19–49.19	0.032
46–55	2.91	0.44–19.4	0.269
56–65	11.01	1.02–119.46	0.049
> 65	29.83	1.75–508.3	0.019
Gender			
Men	Ref		
Women	0.64	0.23–1.76	0.384
Marital status			
Single	Ref		
Married	0.8	0.19–3.38	0.765
Divorced/Separated	1.22	0.2–7.32	0.832
Widow	0.24	0.03–2.05	0.191
Living condition			
Alone	Ref		
Living with someone	1.1	0.16–7.55	0.919
Education level			
Uneducated	Ref		
High school or below	0.49	0.1–2.42	0.38
Bachelor’s degree	1.74	0.28–10.75	0.553
Master’s or PhD	2.48	0.24–26.02	0.448
Employment status			
Unemployed	Ref		
Employed	1.46	0.48–4.51	0.506
Retired	0.76	0.16–3.56	0.727
Mood disorder			
Depressive	Ref		
Bipolar	1.25	0.46–3.42	0.661
Duration of illness (years)			
< 1	Ref		
1–5	0.14	0.02–0.86	0.034
6–10	0.13	0.02–0.86	0.034
> 10	0.22	0.03–1.38	0.106
Number of psychiatric ward admissions	0.9	0.55–1.47	0.672
Positive history of using illegal substances/drugs	0.18	0.04–0.8	0.024
Number of regular medications taken for your mental health	0.66	0.44–0.99	0.044
Positive history of buying your own psychiatric medication			
Always	Ref		
Sometimes	0.38	0.08–1.77	0.217
Never	0.42	0.08–2.12	0.296
Feeling shame about taking psychiatric medication	0.21	0.09–0.52	0.001
Support from family and friends regarding mental illness			
I feel very supported.	Ref		
I feel supported to some extent.	0.69	0.28–1.7	0.416
I don’t feel supported.	0.96	0.28–3.26	0.95
Positive history of chronic diseases	0.72	0.31–1.67	0.45
Positive history of using alternative methods to treat your mental illness	0.59	0.25–1.37	0.217
TSQM global satisfaction score	1.04	1.02–1.06	< 0.001
PDRQ score	0.98	0.93–1.04	0.5

Statistical significance was set at *p*-value < 0.05. Wide CIs in some categories reflect small sample sizes. Nagelkerke *R* square = 0.406.

*OR*, odds ratio; *CI*, confidence interval.

Additional factors negatively associated with adherence included history of illegal substance use (OR = 0.18, 95% CI: 0.04–0.8; *p* = 0.024), a higher number of psychiatric medications (OR = 0.66 per additional medication, 95% CI: 0.44–0.99; *p* = 0.044), and medication-related shame (OR = 0.21, 95% CI: 0.09–0.52; *p* = 0.001). TS (TSQM global score) was positively associated with adherence, with each unit increase corresponding to a 4% higher odds of adherence (OR = 1.04, 95% CI: 1.02–1.06; *p* < 0.001).

Multivariable ordinal logistic regression (**Table 7**) identified education, illness duration, social support, and PDR as independent correlates of satisfaction. Higher educational attainment was positively associated with satisfaction, with patients holding bachelor’s degrees having fivefold higher odds of greater satisfaction compared with those without formal education (OR = 5.04, 95% CI: 1.73–14.66; *p* = 0.003). Patients with an illness duration exceeding 10 years also demonstrated higher odds of greater satisfaction (OR = 6.07, 95% CI: 1.8–20.48; *p* = 0.004).

**Table 7:** Multiple ordinal logistic regression analysis for factors associated with treatment satisfaction levels.

Item	Multivariable analysis		
	Adjusted OR	95% CI	*p*-value
Age (years)			
18–25	Ref		
26–35	0.87	0.35–2.2	0.775
36–45	0.7	0.22–2.2	0.543
46–55	0.74	0.22–2.51	0.627
56–65	2.18	0.51–9.26	0.293
> 65	1.33	0.28–6.43	0.72
Gender			
Men	Ref		
Women	1.07	0.58–1.98	0.827
Marital status			
Single	Ref		
Married	1.49	0.6–3.71	0.389
Divorced/Separated	2.13	0.73–6.24	0.168
Widow	0.69	0.19–2.52	0.578
Living condition			
Alone	Ref		
Living with someone	1.5	0.43–5.15	0.523
Education level			
Uneducated	Ref		
High school or below	2.6	1–6.75	0.049
Bachelor’s degree	5.04	1.73–14.66	0.003
Master’s or PhD	3.21	0.84–12.3	0.089
Employment status			
Unemployed	Ref		
Employed	1.1	0.56–2.19	0.779
Retired	0.45	0.17–1.19	0.107
Mood disorder			
Depressive	Ref		
Bipolar	0.65	0.34–1.26	0.203
Duration of illness (years)			
< 1	Ref		
1–5	2.75	0.81–9.36	0.107
6–10	2.97	0.83–10.55	0.093
> 10	6.07	1.8–20.48	0.004
Number of psychiatric ward admissions	0.95	0.71–1.28	0.745
Positive history of using illegal substances/drugs	0.86	0.31–2.37	0.766
Number of regular medications taken for your mental health	0.84	0.61–1.14	0.254
Positive history of buying your own psychiatric medication			
Always	Ref		
Sometimes	0.58	0.26–1.32	0.195
Never	1.18	0.49–2.83	0.713
Feeling shame about taking psychiatric medication	0.66	0.35–1.22	0.183
Support from family and friends regarding mental illness			
I feel very supported.	Ref		
I feel supported to some extent.	0.52	0.3–0.92	0.024
I don’t feel supported.	0.51	0.23–1.09	0.082
Positive history of chronic diseases	1.15	0.68–1.95	0.595
Positive history of using alternative methods to treat your mental illness	1.02	0.62–1.69	0.94
PDRQ score	1.11	1.07–1.15	< 0.001

Statistical significance was set at *p*-value < 0.05. Nagelkerke *R* square = 0.341.

*OR*, odds ratio; *CI*, confidence interval.

Limited social support was associated with reduced odds of higher satisfaction (OR = 0.52, 95% CI: 0.3–0.92; *p* = 0.024 for some *vs*. strong support). The PDR quality score indicated a strong positive association with satisfaction, with each unit increase corresponding to an 11% higher odds of greater satisfaction (OR = 1.11, 95% CI: 1.07–1.15; *p* < 0.001).

Multivariable ordinal logistic regression (**Table 8**) revealed that medication cost burden, social support, and use of alternative treatments were significantly associated with PDR. Patients who sometimes or never purchased their own psychiatric medication had significantly higher odds of better PDR (OR = 2.96, 95% CI: 1.18–7.44; *p* = 0.021 and OR = 5.04, 95% CI: 1.81–14.06; *p* = 0.002, respectively) compared with those who always bore medication costs.

**Table 8:** Multiple ordinal logistic regression analysis for factors associated with patient–doctor relationship levels.

Item	Multivariable analysis		
	Adjusted OR	95% CI	*p*-value
Age (years)			
18–25	Ref		
26–35	0.54	0.18–1.6	0.269
36–45	0.53	0.13–2.13	0.372
46–55	0.53	0.12–2.35	0.406
56–65	0.3	0.05–1.7	0.174
> 65	0.33	0.05–2.14	0.243
Gender			
Men	Ref		
Women	1.13	0.55–2.32	0.742
Marital status			
Single	Ref		
Married	1.14	0.39–3.38	0.809
Divorced/Separated	1.79	0.48–6.74	0.389
Widow	1.27	0.25–6.56	0.773
Living condition			
Alone	Ref		
Living with someone	0.99	0.22–4.45	0.992
Education level			
Uneducated	Ref		
High school or below	0.67	0.19–2.3	0.521
Bachelor’s degree	0.3	0.08–1.13	0.075
Master’s or PhD	0.21	0.04–1.05	0.058
Employment status			
Unemployed	Ref		
Employed	1.92	0.87–4.23	0.108
Retired	1.69	0.56–5.1	0.354
Mood disorder			
Depressive	Ref		
Bipolar	0.72	0.34–1.54	0.395
Duration of illness (years)			
< 1	Ref		
1–5	0.51	0.11–2.46	0.405
6–10	0.21	0.04–1.09	0.063
> 10	0.57	0.12–2.74	0.479
Number of psychiatric ward admissions	1.01	0.71–1.45	0.944
Positive history of using illegal substances/drugs	0.49	0.16–1.48	0.204
Number of regular medications taken for your mental health	0.81	0.56–1.17	0.266
Positive history of buying your own psychiatric medication			
Always	Ref		
Sometimes	2.96	1.18–7.44	0.021
Never	5.04	1.81–14.06	0.002
Feeling shame about taking psychiatric medication	0.52	0.26–1.06	0.072
Support from family and friends regarding mental illness			
I feel very supported.	Ref		
I feel supported to some extent.	0.37	0.19–0.7	0.002
I don’t feel supported.	0.34	0.14–0.85	0.021
Positive history of chronic diseases	1.09	0.58–2.03	0.797
Positive history of using alternative methods to treat your mental illness	2.23	1.21–4.11	0.01

Statistical significance was set at *p*-value < 0.05. Nagelkerke *R* square = 0.249.

*OR*, odds ratio; *CI*, confidence interval.

Both limited and absent social support were associated with lower odds of better PDR (OR = 0.37; 95% CI: 0.19–0.7; *p* = 0.002 and OR = 0.34; 95% CI: 0.14–0.85; *p* = 0.021, respectively). Patients who used alternative treatment methods had higher odds of better PDR (OR = 2.23; 95% CI: 1.21–4.11; *p* = 0.01) than those who did not.

## 4 Discussion

The goals of our study were to assess MA, TS, and the quality of PDR in individuals suffering from mood disorders, identifying key sociodemographic and clinical factors that could influence these interconnected outcomes. In our study, over three-quarters of the participants displayed good adherence to their medication, higher than reported in a study conducted at the Amal Psychiatric Outpatient Clinic in Jazan, Saudi Arabia, which found that about 60% of schizophrenic patients had a high adherence level (37). Another study conducted at outpatient clinics at the Psychological Care Department at King Fahad Medical City, Riyadh, Saudi Arabia, found that 44.5% of schizophrenic patients exhibited good adherence (51). A probable explanation for this discrepancy is that our study included mood disorders, whereas the others focused on patients with psychotic disorders—a condition typically marked by greater chronicity, cognitive impairment, and higher adverse-effect burden, all of which are associated with poorer adherence (52). Despite our findings, it is crucial to monitor MA closely while caring for mood-disorder patients and to support adherence by various means. For instance, two studies revealed that tailored psychoeducation helps patients build insight and understand the value of their medication, which is strongly linked to better adherence (53, 54). Furthermore, a systematic review and meta-analysis found that simple strategies, such as brief teaching sessions, motivational interviewing, and even text messages, can make daily treatment feel more manageable and improve adherence across settings (55). Keeping medication regimens simple can also help patients remain adherent (56). Nonetheless, proactively addressing side effects is essential, as adverse reactions remain a major reason patients stop taking medication (57).

In our study, the level of satisfaction varied, with about one-third of the participants having low satisfaction and about one-quarter having very high satisfaction. In a Saudi study that included psychiatric inpatients and outpatients diagnosed with schizophrenia and receiving psychotropic medication, over half (58.3%) of the patients said they were satisfied (58). Variation across studies may stem from differences in service structure and patient experience, such as the quality of interaction with clinical staff, ease of access to care, waiting time, and the perceived effectiveness of treatment (59). Differences in these factors between healthcare sites could contribute to differences in levels of satisfaction between studies. Therefore, further Saudi studies are needed to explore the possible reasons for treatment dissatisfaction and to develop means to mitigate it.

The majority of participants in our study (62.31%) reported good PDR (PDRQ-9 score ≥ 36), which is lower than the estimate from a study in Thailand among psychiatric outpatients with depression (82.2%). That study suggested that strong PDR, supported by good knowledge and positive attitudes toward major depressive disorder, helps patients feel more accepted and supported by their healthcare providers and the community (47). This difference may be shaped by how care is delivered and the consistency of follow-up, as patients place high value on physician competence, clear communication, involvement in decisions, and continuity of care, all of which strengthen PDR (60). Cultural expectations, including norms regarding respect, gender roles, family involvement, and spiritual beliefs, can also influence how patients perceive and evaluate their interactions with clinicians (61). Clinically, strengthening PDR may be supported by providing time and space for patient–clinician conversations, supporting shared decision-making, ensuring continuity of care, and involving family members or caregivers when appropriate (62).

We found that good MA was strongly associated with higher TS and a better PDR. We also observed that higher satisfaction was linked to better PDR. This pattern indicates that adherence, satisfaction, and PDR mutually reinforce one another; improvement in one domain may positively influence the others, a finding supported by multicenter research on patients with bipolar disorder, which indicated that these three factors are closely interconnected (63). These findings are also consistent with another study, which found that stronger physician communication significantly improves patient adherence to treatment in psychiatric populations (64). Similarly, a systematic review revealed that a better PDR—including clear communication and collaboration—was associated with higher treatment adherence in mental healthcare (65). This point highlights how important it is to build trust and good communication between doctors and patients. We therefore recommend regularly checking patients’ satisfaction and their relationship with their doctors as part of routine care so that those at risk of discontinuing their medication can be identified and supported early.

In our study, TS emerged as a contributing factor of adherence; an increase in the TSQM global satisfaction score was associated with an increase in the odds of MA. Our finding echoes previous research conducted at Al Amal Psychiatric Hospital in Riyadh, which found that adherence to antidepressants was associated with TS with the antidepressants (31). Additionally, a cross−sectional study conducted among hypertensive patients found that TS was significantly and strongly associated with adherence to antihypertensive medication (66). Furthermore, the quality of the PDR—quantified by the PDRQ—was positively correlated with satisfaction in our study; an increase in the PDRQ score was associated with an increase in the odds of being more satisfied. This finding is consistent with a systematic review of adolescent and adult psychiatric patients with various diagnoses (schizophrenia, bipolar, and depressive disorders) (67). We recommend that doctors communicate with empathy, address patients’ concerns, and involve them in decisions, so patients feel more satisfied and become more likely to adhere to treatment.

According to our multivariable logistic regression analysis, the 36–45-, 56–65-, and > 65-year-old groups had significantly higher odds of adherence than the 18–25-year-old group. This pattern aligns with a systematic review and meta-analysis among psychiatric patients that found adherence peaks in mid-life, between 35 and 65 years (68). Our finding also aligns with those of a study from the Ayder Referral Hospital in Mekelle, Ethiopia, conducted among psychiatric patients, including schizophrenia, mood disorders, and other diagnoses; the study’s results indicated that older adults demonstrated higher adherence levels (69). Based on these results, it is important to provide younger patients with additional support—such as psychoeducation—to help them maintain medication adherence and reduce their risk of nonadherence. For instance, a study conducted in Iran found that psychoeducation was an effective intervention for improving MA in patients with bipolar disorder (54).

We found a relationship between adherence and marital status: married individuals had higher rates of good adherence and lower rates of poor adherence than single individuals. This finding is consistent with a study conducted at the Institute of Psychiatry, Ain Shams University, Egypt, that emphasized that marriage is positively correlated with MA among bipolar patients (70). However, our finding contradicts a study conducted across nine specialized mental health centers of the FondaMental Foundation in France, which involved 382 patients diagnosed with bipolar disorder and found no association between marital status and adherence (71). We suggest that the effect of marital status on adherence may vary depending on cultural or community background. Future research in Saudi Arabia should explore how marital status—and the roles and responsibilities associated with it—affects adherence among psychiatric patients. Though it was not among psychiatric patients, a prospective study in the USA among heart-failure patients found that married individuals were more likely to report having someone reminding them to take their prescribed medication and assisting them in doing so (72).

Our study demonstrated that education level was associated with both TS and the quality of the PDR. Relative to individuals with no formal education, patients with a high school education or lower exhibited higher odds of reporting greater satisfaction, whereas those with a bachelor’s degree demonstrated even higher odds. Similarly, bachelor-level patients were significantly more likely to describe a favorable PDR than participants with lower educational attainment. These findings are consistent with previous research, including a study of 127 psychiatric inpatients in Germany, which found that higher education was associated with improved adherence and satisfaction (73). Another study conducted in Europe among primary-care patients found that higher education predicted a more positive PDR (74). These findings emphasize the importance of physicians adapting their communication to patients’ education levels, ultimately enhancing adherence, satisfaction, and the PDR. This recommendation is supported by a clinical trial conducted in Iran among hypertensive patients, which demonstrated that physician communication training provided multiple benefits, including MA (75).

In our study, longer illness duration was associated with contrasting outcomes. Patients living with chronic mental health conditions reported significantly greater TS and better PDR. This finding aligns with evidence that continuity of care and repeated interactions with clinicians can foster trust, improve communication, and strengthen the therapeutic alliance (76). Similarly, a Spanish study demonstrated that structured psychoeducation significantly increased patients’ illness-related knowledge, which improved treatment adherence and acceptance of clinical recommendations (77). However, in our sample, extended duration was also associated with significantly lower odds of MA, consistent with findings from an Ethiopian cohort of patients with schizophrenia, where longer illness duration predicted nonadherence (78). Nevertheless, our study contrasts with a South Korean study of major depressive disorder, in which treatment continuity improved with longer illness duration, revealing the complexity of how chronicity influences adherence (79). Possible explanations for these differences include treatment fatigue, evolving perceptions of illness and cure, and emergent treatment resistance over time (78). Clinically, these observations highlight the dual imperative of nurturing long-term, trust-based therapeutic relationships while instituting rigorous adherence monitoring and individualized support, particularly for patients with longstanding psychiatric conditions, so the benefits of a strong alliance are not undermined by declining medication engagement. A reasonable hypothesis is that, over time, patients become increasingly familiar with their clinicians and the overall treatment process, which may strengthen trust and communication. However, remaining in treatment for many years might also lead to reduced motivation or treatment fatigue. This combination may help explain why satisfaction increases while adherence declines among patients with longer illness duration. Evidence supporting this hypothesis has been reported across multiple clinical settings. For example, a study among chronic psychiatric populations found that familiarity with the treatment team enhanced perceived alliance and dependability (76). Conversely, reviews and expert guidelines have noted that factors such as recurrent episodes, multiple medication trials, and ongoing symptom burden are associated with poorer adherence over time, often reflecting manifestations of treatment fatigue (52, 80, 81).

Patients in our study who accepted they had a mental illness and needed treatment were more likely to be satisfied. This finding is consistent with a study in the UK involving patients with bipolar disorder, which provided qualitative data on the relationship between patients’ beliefs about medication and adherence behavior (82). That study found that insight was highly correlated with better adherence and patient–doctor trust (82). Such insight likely leads to greater motivation, trust in clinicians, and openness to treatment (83). We recommend that future Saudi researchers assess whether structured psychoeducation or motivational interviewing can improve insight and, in turn, satisfaction. Our finding—that patients with better insight reported higher satisfaction—aligns with evidence that patients’ beliefs about their illness influence their engagement. This pattern in our results reinforces the broader observation that patients’ perceptions of their illness strongly shape their motivation and treatment behavior. Evidence from Saudi Arabia supports this observation as well. For example, a recent study of outpatients with schizophrenia found that most patients demonstrated fair-to-good insight and moderate levels of MA, and that better insight was significantly associated with higher adherence (84). Patients with poorer insight tended to exhibit lower adherence and more prominent symptom severity, highlighting the potential importance of improving insight in sustaining long-term engagement with treatment (84). In addition, the study emphasized that regularly assessing patients’ insight is essential for identifying those at higher risk of nonadherence and for guiding individualized interventions to enhance treatment participation (84).

Our study demonstrated that patients with a history of illegal substance use had significantly lower odds of adherence compared with those without such a history. These findings align with a systematic review and meta-analysis among individuals with mental illnesses, which identified a strong association between substance use and medication nonadherence (10). Similar conclusions have been reported in other studies; for instance, research among psychiatric outpatients found that even intermittent substance use disrupts adherence and increases the probability of treatment discontinuation (85). Substance use can interfere with daily routines and impair decision-making, making consistent medication intake more challenging (52). These results emphasize the importance of routinely screening for substance use and implementing early interventions.

In our study, each additional regular psychiatric medication was associated with a lower probability of adherence. This pattern has been consistently documented in both international and regional research. An Omani study (86) found that complex regimens significantly reduce adherence, and other studies have reported similar findings. For example, a multicenter European study indicated that polypharmacy and increased regimen complexity were associated with lower MA (73). As patients take more medications, the treatment plan becomes more difficult to follow and may feel more burdensome, contributing to reduced adherence. Future research in Saudi Arabia could explore whether simplifying medication regimens—such as minimizing unnecessary polypharmacy, consolidating dosing schedules, or implementing periodic medication reviews—can improve long-term adherence among patients.

Patients in our study who never bought their own medication had significantly higher odds of reporting a better relationship with their physicians. One possible explanation for this finding is that financial relief, coupled with assured access to medication, fosters a sense of trust and, therefore, may be associated with a stronger PDR. This finding aligns with a previous study conducted in Ethiopia among psychiatric patients, which identified medication availability as a significant predictor of greater patient satisfaction (87). Another study conducted in the USA among the general population found that high medical cost burdens are linked to lower fiduciary trust in physicians and more negative perceptions of care (88). This finding is also consistent with earlier research in which psychiatric outpatients with lower involvement in treatment decision-making reported greater reliance on their healthcare providers and higher trust and confidence in them (89). These findings are further supported by two studies conducted in Saudi Arabia, the first of which involved psychiatric outpatients undergoing antidepressant treatment in Riyadh. The study found that higher TS was significantly associated with better adherence, illustrating that satisfaction with care—including confidence in continuous access to medication—strengthens the therapeutic relationship (31). This observation regarding the link between access-related satisfaction and improved adherence aligns with our finding that assured access to medication fosters a more positive therapeutic PDR. Another study, conducted among the general population using the e-prescription system across Saudi primary-care settings, reported high levels of satisfaction with medication availability, indicating that reliable access to prescribed medication promotes more positive perceptions of care (90). These findings collectively support the idea that satisfaction driven by constant medication availability enhances trust and overall care experience, further reinforcing our conclusion that uninterrupted access to medication improves both patient satisfaction and the therapeutic alliance. Therefore, ensuring consistent and free access to medication may strengthen both patient satisfaction and the therapeutic relationship.

We found that patients who felt shame about their medication had significantly lower odds of adherence than those who did not experience shame. The good-adherence group included a significantly lower proportion of patients reporting shame about taking medication compared with the poor-adherence group. Our findings align with previous research, including an Ethiopian study that identified self-stigma as a key factor negatively influencing MA and prompting voluntary discontinuation of psychiatric medication (91). A narrative review by Pompili et al. (2009) further concluded—across multiple studies with diverse samples—that internalized stigma significantly reduces both adherence and TS (92). Additionally, in our study, a significant difference was observed among groups regarding feelings of shame about taking psychiatric medication and the quality of the PDR. A study in Germany demonstrated that self-stigma and shame among psychiatric populations negatively affect communication with healthcare providers, reducing the likelihood that patients express their views openly during consultations (93). This pattern indicates that shame can erode trust and openness, creating barriers to care, a finding further supported by a study in the USA involving patients diagnosed with depression, schizophrenia, and bipolar disorder (94). That review showed that self-stigma and shame substantially reduced patients’ trust in their clinicians and limited their willingness to communicate openly during treatment interactions. These observations—particularly the evidence that self-stigma and shame undermine trust in clinicians—are consistent with our findings, which indicate that shame significantly compromises the quality of the PDR. Clinically, these findings underscore the need for healthcare providers to proactively address self-stigma and feelings of shame through psychoeducation, empathetic communication, and a nonjudgmental clinical environment, thereby enhancing adherence, PDR quality, and overall TS.

The present study identified perceived social support from family and friends as an influential factor regarding TS and the therapeutic alliance; participants who felt highly supported reported the highest satisfaction and were more likely to describe a good PDR. These results align with a cross-sectional study among patients with schizophrenia that emphasized the importance of perceived social support from loved ones in patients’ overall TS (95). Social support may be associated with a better treatment experience, as it reassures patients, improves adherence behaviors, and reinforces the value of clinical care, thereby deepening trust between patients and clinicians. These findings are supported by a study conducted in Turkey involving patients with schizophrenia, which found that the higher the level of perceived family support, the better the treatment adherence and engagement in healthcare (96). These results are consistent with our study, emphasizing that support from loved ones strengthens the therapeutic alliance and enhances TS. Collectively, this evidence underscores the clinical importance of routinely assessing patients’ social support and, when appropriate, actively involving family members or other key caregivers to enhance satisfaction, strengthen therapeutic relationships, and foster better adherence.

This study found that patients who employed alternative or culturally familiar strategies (such as prayer, herbal remedies, or physical activity, either alone or alongside psychiatric medication) had significantly higher odds of reporting better PDR than those who did not use such methods. This finding is consistent with the results of a study conducted in South Korea using a large outpatient sample. In that study, traditional Korean medicine users reported differential levels of satisfaction with their medical doctors compared with nonusers, suggesting that the use of culturally familiar healing practices might influence—or even enhance—the perceived quality of the PDR (97). Although not specifically addressing alternative or culturally familiar strategies, a study in Zimbabwe involving 573 ethnically diverse primary-care patients found that receiving structured problem-solving therapy, a form of alternative strategy—was associated with stronger therapeutic alliances than receiving usual care (98). Our finding, however, partially diverges from a Mexican study among rheumatoid arthritis outpatients, which found no overall difference in PDR between users and nonusers of alternative medicine, although users who disclosed their practices reported greater satisfaction with treatment and a shared understanding of symptom causes (99). One plausible explanation for this discrepancy is that patients in our sample may have felt sufficiently comfortable disclosing their alternative practices, fostering openness and trust. These observations emphasize the importance of clinicians eliciting and acknowledging patients’ health beliefs and culturally rooted coping strategies, which can enhance the therapeutic relationship and overall treatment experience. A study conducted in Riyadh, involving adult psychiatric patients with various psychiatric illnesses, indicated that psychiatric patients commonly use complementary and alternative medicine (CAM), including spiritual therapies, herbal remedies, and exercise, with the majority reporting satisfaction (100). Although the study did not directly measure PDR, the high prevalence and positive perception of CAM suggest that culturally familiar strategies may influence trust, openness, and engagement with clinicians (100). Future Saudi-based studies should investigate how the disclosure and integration of alternative practices influence therapeutic alliances across various clinical and cultural contexts.

Finally, our study found that, compared with the poor-adherence group, the good-adherence group had significantly lower percentages of patients who perceived alternative methods as more effective than psychiatric medication. Furthermore, perceiving alternative methods as more effective was associated with lower treatment satisfaction. Evidence from the Federal Neuropsychiatric Hospital in Maiduguri, Nigeria, indicated that seeking traditional and spiritual methods increased the odds of poor adherence more than sixfold (101). Similarly, a hospital-based study in Saudi Arabia found that most psychiatric patients (82.2%) reported using at least one form of complementary or alternative therapy, ranging from spiritual healing and Qur’an recitation to cupping (hijama), relaxation techniques, or herbal remedies, with nearly half of the patients not sharing this information with their psychiatrists (100). Another Saudi study reported that individuals with psychiatric disorders are more likely than nonpsychiatric controls to seek faith-healing services (102). Collectively, these findings underscore the importance of clinicians exploring and addressing misconceptions about alternative therapies through culturally sensitive education, thereby enhancing adherence, satisfaction, and trust in psychiatric care.

## 5 Strengths and limitations

This study has several strengths, as it employed well-established and validated tools (MARS-10, TSQM-1.4, and PDRQ-9), adding credibility to the results. The topic has been relatively underexplored in the local Saudi context, making the findings particularly relevant to psychiatric care in Saudi Arabia. Additionally, the study included a sufficiently large sample, which enabled the identification of statistically significant findings.

Nevertheless, some limitations are worth noting. First, as our study is cross-sectional, it cannot establish cause-and-effect relationships between variables. Future Saudi research would benefit from longitudinal designs to better understand how these relationships evolve. The second limitation is that the study was conducted in only one hospital, which may not reflect the experiences of patients in other Saudi healthcare facilities or regions. To obtain a more comprehensive picture, future Saudi studies should include participants from multiple centers and diverse geographic areas. A third limitation is that the data-collection method, relying on telephone and WhatsApp, may have resulted in the underrepresentation, to some extent, of older persons or individuals with restricted access to, or proficiency in, these technologies, constraining the generalizability of our findings to such individuals. We recommend that future studies employ more rigorous modes of data collection, such as in-person interviews, to ensure more inclusive participation and enhance the representativeness of the sample. A fourth limitation is that the reliance on self-reported data introduces the potential for recall and social desirability biases, particularly regarding sensitive issues, such as stigma. Incorporating more objective data sources, such as medication refill records, could strengthen the validity of future findings. Fifth, MA was assessed in our study using self-reported measures only, with no objective indicators, such as pill counts or pharmacy refill records. Therefore, we recommend that future Saudi studies incorporate both self-reported and objective measures of adherence to provide a more robust and comprehensive assessment. A sixth limitation is that some particular variables, such as perceived ease of obtaining medication, were assessed using subjective items without operational definitions. This factor may limit the precision and reproducibility of such measures. Therefore, we recommend that future studies employ clearly defined, validated measures to assess such variables more rigorously. A seventh limitation is the wide confidence intervals observed for certain age groups in the multivariable analysis, particularly for participants aged > 65 years old, reflecting sparse-data bias due to smaller sample sizes in these categories. Although this aspect does not invalidate the direction of the association, these estimates should be interpreted with caution. Finally, despite collecting detailed sociodemographic, psychiatric, and medication-related information, our study did not assess cultural or religious beliefs, which may meaningfully influence participants’ experiences and adherence-related behavior. Examining these factors in future Saudi studies could enrich the interpretation and contextualization of the findings. Future studies should incorporate cultural and religious variables and examine how they interact with other sociodemographic and clinical factors to provide a more comprehensive understanding.

## 6 Conclusions

Our study assessed MA, TS, and the quality of PDR in individuals with mood disorders, identifying key sociodemographic and clinical determinants that influence these interconnected outcomes. Most participants (79.2%) exhibited good MA; however, approximately one-third (34.2%) exhibited low satisfaction with their treatment, while nearly two-thirds (62.3%) reported good relationships with their doctors. The MA was positively correlated with older age, being married, and increased TS, and it was negatively influenced by extended illness duration, illicit substance use, medication-related self-stigma, and perceiving alternative methods as more effective than psychiatric medication. Higher TS was evident among patients who acknowledged their illness, were educated, had lived with their mood disorder for 10 years or more, had strong family and social support, and reported a robust PDR. Conversely, satisfaction decreased with limited social support and a preference for alternative therapies. The probability of a better PDR increased with higher education, longer illness duration, not having to purchase psychiatric medication personally, and using alternative methods to treat the mental illness, but this probability decreased markedly with minimal social support. Nonetheless, MA, TS, and a positive PDR were mutually reinforcing, emphasizing their interdependence.

Our findings collectively emphasize the importance of addressing modifiable factors, such as medication-related stigma, social and family support, psychoeducation, and patient–clinician communication to enhance mood-disorder outcomes. Clinicians and healthcare systems should develop and implement targeted interventions, such as structured stigma-reduction programs, family-based support initiatives, streamlined pharmacological strategies to reduce polypharmacy, routine screening for substance use, and continuous patient education.

## Data Availability

The raw data supporting the conclusions of this article will be made available by the authors, without undue reservation.
