# Supplementary material for: Medication adherence, treatment satisfaction, and patient–doctor relationship in patients with mood disorders at a Saudi tertiary care hospital: a cross-sectional study
Source: Front Psychiatry. 2026 Jan 16;16:1695769. doi: 10.3389/fpsyt.2025.1695769 (PMC12856496; doi:10.3389/fpsyt.2025.1695769)
Supplement: Supplementary file 1 [file Table1.pdf]

Supplementary Material

Supplementary Table 1. MARS-10 scale results of the studied patients.

Item	Total patients (*n*=260)
Do you ever forget to take your medication?	
No	87 (33.46%)
Yes	173 (66.54%)
Are you careless at times about taking your medication?	
No	177 (68.08%)
Yes	83 (31.92%)
When you feel better, do you sometimes stop taking your medication?	
No	201 (77.31%)
Yes	59 (22.69%)
Sometimes, if you feel worse when you take the medication, do you stop taking it?	
No	208 (80%)
Yes	52 (20%)
I take my medication only when I am sick.	

No	215 (82.69%)
Yes	45 (17.31%)
It is unnatural for my mind and body to be controlled by medication.	
No	147 (56.5%)
Yes	113 (43.5%)
My thoughts are clearer on medication.	
No	49 (18.85%)
Yes	211 (81.15%)
By staying on medication, I can prevent getting sick.	
No	47 (18.08%)
Yes	213 (81.92%)
I feel weird, like a zombie, on medication.	
No	219 (84.23%)
Yes	41 (15.77%)
Medication makes me feel tired and sluggish.	
No	133 (51.15%)
Yes	127 (48.85%)

Total score	6.96 ± 2.05
Adherence	
Poor (score 0–5)	54 (20.8%)
Good (score 6–10)	206 (79.2%)

Numerical data are presented as mean ± SD, categorical data as frequency (%).

Supplementary Table 2. TSQM-1.4 scale results of the studied patients.

Item	Total patients (*n*=260)
➢ Effectiveness domain	
How satisfied or dissatisfied are you with the ability of the medication to prevent or treat your condition?	
Extremely dissatisfied	6 (2.31%)
Very dissatisfied	8 (3.08%)
Dissatisfied	9 (3.46%)
Somewhat satisfied	56 (21.54%)
Satisfied	77 (29.62%)
Very satisfied	49 (18.85%)
Extremely satisfied	55 (21.15%)

How satisfied or dissatisfied are you with the way the medication relieves your symptoms?	
Extremely dissatisfied	7 (2.69%)
Very dissatisfied	5 (1.92%)
Dissatisfied	22 (8.46%)
Somewhat satisfied	56 (21.54%)
Satisfied	74 (28.46%)
Very satisfied	39 (15%)
Extremely satisfied	57 (21.92%)
How satisfied or dissatisfied are you with the amount of time it takes the medication to start working?	
Extremely dissatisfied	9 (3.46%)
Very dissatisfied	11 (4.23%)
Dissatisfied	24 (9.23%)
Somewhat satisfied	57 (21.92%)
Satisfied	79 (30.38%)
Very satisfied	35 (13.46%)
Extremely satisfied	45 (17.31%)
Effectiveness score	66.62 ± 21.79

As a result of taking this medication, do you experience any side effects at all?	
No	128 (49.23%)
Yes	132 (50.77%)
➢ Side effects domain	**(*n*=132)**
How bothersome are the side effects of the medication you take to treat your condition?	
Extremely bothersome	22 (16.67%)
Very bothersome	32 (24.24%)
Somewhat bothersome	54 (40.91%)
A little bothersome	21 (15.91%)
Not at all bothersome	3 (2.27%)
To what extent do the side effects interfere with your physical health and ability to function (strength, energy levels, etc.)?	
A great deal	28 (21.21%)
Quite a bit	46 (34.85%)
Somewhat	31 (23.48%)
Minimally	16 (12.12%)
Not at all	11 (8.33%)

To what extent do the side effects interfere with your mental function (ability to think clearly, stay awake, etc.)?	
A great deal	21 (15.91%)
Quite a bit	38 (28.79%)
Somewhat	40 (30.3%)
Minimally	11 (8.33%)
Not at all	22 (16.67%)
To what degree have medication side effects affected your overall satisfaction with the medication?	
A great deal	18 (13.64%)
Quite a bit	41 (31.06%)
Somewhat	41 (31.06%)
Minimally	24 (18.18%)
Not at all	8 (6.06%)
Side effects score	41.71 ± 21.71
➢ Convenience domain	**(*n*=260)**
How easy or difficult is it to use the medication in its current form?	
Extremely difficult	3 (1.15%)

Very difficult	5 (1.92%)
Difficult	6 (2.31%)
Somewhat easy	28 (10.77%)
Easy	70 (26.92%)
Very easy	50 (19.23%)
Extremely easy	98 (37.69%)
How easy or difficult is it to plan when you use the medication each time?	
Extremely difficult	3 (1.2%)
Very difficult	5 (1.9%)
Difficult	10 (3.8%)
Somewhat easy	54 (20.8%)
Easy	58 (22.3%)
Very easy	46 (17.7%)
Extremely easy	84 (32.3%)
How convenient or inconvenient is it to take the medication as instructed?	
Extremely inconvenient	2 (0.77%)
Very inconvenient	1 (0.38%)

Inconvenient	3 (1.15%)
Somewhat convenient	43 (16.54%)
Convenient	82 (31.54%)
Very convenient	45 (17.31%)
Extremely convenient	84 (32.31%)
Convenience score	76.18 ± 19.44
➢ Global satisfaction domain	
Overall, how confident are you that taking this medication is a good thing for you?	
Not at all confident	10 (3.85%)
A little confident	21 (8.08%)
Somewhat confident	60 (23.08%)
Very confident	76 (29.23%)
Extremely confident	93 (35.77%)
How certain are you that the good things about your medication outweigh the bad things?	
Not at all certain	17 (6.54%)
A little certain	27 (10.38%)
Somewhat certain	60 (23.08%)

Very certain	79 (30.38%)
Extremely certain	77 (29.62%)
Taking all things into account, how satisfied or dissatisfied are you with this medication?	
Extremely dissatisfied	8 (3.08%)
Very dissatisfied	2 (0.77%)
Dissatisfied	9 (3.46%)
Somewhat satisfied	51 (19.62%)
Satisfied	78 (30%)
Very satisfied	45 (17.31%)
Extremely satisfied	67 (25.77%)
Global satisfaction score	69.69 ± 24.52
Very low satisfaction (score <40)	24 (9.23%)
Low satisfaction (score 40–64)	89 (34.23%)
Moderate satisfaction (score 65–79)	56 (21.54%)
High satisfaction (score 80–89)	21 (8.08%)
Very high satisfaction (score >90)	70 (26.92%)

Numerical data are presented as mean ± SD, categorical data as frequency (%).

Supplementary Table 3. PDRQ-9 scale results of the studied patients.

Item	Total patients (*n*=260)
My primary physician helps me.	
Strongly agree	124 (47.69%)
Agree	98 (37.69%)
Neutral	18 (6.92%)
Disagree	10 (3.85%)
Strongly disagree	10 (3.85%)
My primary physician has enough time for me.	
Strongly agree	101 (38.85%)
Agree	91 (35%)
Neutral	35 (13.46%)
Disagree	21 (8.08%)
Strongly disagree	12 (4.62%)
I trust my primary physician.	
Strongly agree	128 (49.23%)

Agree	86 (33.08%)
Neutral	28 (10.77%)
Disagree	9 (3.46%)
Strongly disagree	9 (3.46%)
My primary physician understands me.	
Strongly agree	118 (45.38%)
Agree	78 (30%)
Neutral	42 (16.15%)
Disagree	11 (4.23%)
Strongly disagree	11 (4.23%)
My primary physician is dedicated to help me.	
Strongly agree	119 (45.77%)
Agree	78 (30%)
Neutral	37 (14.23%)
Disagree	15 (5.77%)
Strongly disagree	11 (4.23%)
My primary physician and I agree on the nature of my medical symptoms.	

Strongly agree	106 (40.77%)
Agree	96 (36.92%)
Neutral	43 (16.54%)
Disagree	10 (3.85%)
Strongly disagree	5 (1.92%)
I can talk to my primary physician.	
Strongly agree	117 (45%)
Agree	96 (36.92%)
Neutral	22 (8.46%)
Disagree	15 (5.77%)
Strongly disagree	10 (3.85%)
I feel content with my primary physician's treatment.	
Strongly agree	111 (42.69%)
Agree	99 (38.08%)
Neutral	31 (11.92%)
Disagree	14 (5.38%)
Strongly disagree	5 (1.92%)

I find my primary physician easily accessible.	
Strongly agree	85 (32.69%)
Agree	74 (28.46%)
Neutral	41 (15.77%)
Disagree	33 (12.69%)
Strongly disagree	27 (10.38%)
PDRQ-9 score	36.5 ± 8.08
Poor relationship (score ≤17)	7 (2.69%)
Moderate relationship (score 18–35)	91 (35%)
Good relationship (score ≥36)	162 (62.31%)

Numerical data are presented as mean ± SD, categorical data as frequency (%).
